# SPC25 promotes proliferation and stemness of hepatocellular carcinoma cells via the DNA-PK/AKT/Notch1 signaling pathway

**DOI:** 10.7150/ijbs.71694

**Published:** 2022-08-15

**Authors:** Jieying Yang, Yue Huang, Mengjia Song, Qiuzhong Pan, Jingjing Zhao, Junyi He, Dijun Ouyang, Chaopin Yang, Yulong Han, Yan Tang, Qijing Wang, Jia He, Yongqiang Li, Hao Chen, Desheng Weng, Tong Xiang, JianChuan Xia

**Affiliations:** 1 Department of Biotherapy, Sun Yat-sen University Cancer Center, Guangzhou, China.; 2 Collaborative Innovation Center for Cancer Medicine, State Key Laboratory of Oncology in South China, Sun Yat-sen University Cancer Center, Guangzhou, China.; 3 Department of Oncology, The Fifth Affiliated Hospital of Sun Yat-Sen University, Zhuhai, Guangdong, China.

**Keywords:** SPC25, hepatocellular carcinoma, stemness, DNA-PK/AKT, Notch1

## Abstract

The imbalance of kinetochore-microtubule attachment during cell mitosis is a response to the initiation and progression of human cancers. Spindle component 25 (SPC25) is indispensable for spindle apparatus organization and chromosome segregation. SPC25 plays an important role in the development of malignant tumors, but its role in hepatocellular carcinoma (HCC) is yet to be determined. In this study, we aimed to preliminarily investigate the role of SPC25 in HCC progression and the molecular mechanisms underlying the process. We identified SPC25 as a clinically notable molecule significantly correlated with the grade of malignancy and poor survival in both The Cancer Genome Atlas (TCGA) cohort and the HCC patient cohort from our center. Mechanistically, SPC25 promoted the incidence of DNA damage and activated the DNA-PK/Akt/Notch1 signaling cascade in HCC cells; the NICD/ RBP-Jκ complex directly targeted SOX2 and NANOG in a transcriptional manner to regulate the proliferation and self-renewal of HCC cells. Our study suggests that HCC-intrinsic SPC25/DNA-PK/Akt/Notch1 signaling is an important mechanism to promote carcinogenesis by regulating the proliferation and stemness program, which provides possible biomarkers for predicting HCC progression and poor survival, as well as potential therapeutic targets for HCC patients.

## Introduction

Recent epidemiological data indicates that hepatocellular carcinoma (HCC) is the sixth most commonly occurring cancer and the fourth leading cause of cancer-related deaths worldwide 1. A total of 841 080 HCC cases, which resulted in 781 631 deaths were reported in 2018. China is one of the areas with the highest risk for HCC, the key determinants of which are chronic hepatitis B virus (HBV) infection and aflatoxin exposure 2. However, the underlying mechanism resulting in carcinogenesis and its development is yet to be clarified. Literature reviews in large volumes indicate that some dysregulated genes controlling cell division and proliferation play important roles in hepatocarcinogenesis 3.

SPC25 is one of the four proteins comprising the nuclear division cycle 80 (NDC80) complex, and the other three components are Ndc80p, Nuf2p, spindle component 24 (SPC24). The NDC80 complex is the most highly conserved kinetochore protein for maintaining the integrity of chromosomes and establishing stable kinetochore-microtubule attachment and tension during cell mitosis 4, 5. Various factors contributing to the dysfunction of the complex can lead to abnormal chromosome segregation, which can further affect cell division and ultimately result in abnormal proliferation 6. Recent studies have reported on SPC25 overexpression in various tumors, including lung cancer 7, prostate cancer 8, colorectal cancer, and gastric cancer 9. High expression of SPC25 as a key component of the NDC80 complex leads to enhanced tumor cell proliferation and advanced degree of malignancy by inducing disorganized cell mitosis, further affecting the prognosis of patients with tumors. Moreover, in prostate cancer cells and non-small cell lung adenocarcinoma cells, SPC25 can increase the properties of cancer stem cells (CSCs) and may facilitate the proliferation of tumor cells by promoting the formation of cancer stem-like cells (CSLCs) 7, 8. Several studies on HCC have indicated the association of SPC25 upregulation with the promotion of cell proliferation and poor prognosis in patients with HCC 10-12. However, these studies are too small in number that no reliable conclusion may be deduced on the correlation between SPC25 and HCC. In addition, the definite mechanism that allows SPC25 to regulate HCC progression remains unclear.

Current studies indicate that abnormal chromosome segregation and defects in checkpoint signaling leads to chromosomal instability (CIN) 13, which is one of the hallmarks of numerous cancers. Its occurrence has been reported in many studies in the very early stages of tumorigenesis, suggesting CIN can be a major initiating event in tumor formation 14, 15. These theories provide a feasible mechanism underlying the ability of SPC25 to promote HCC tumorigenesis. In addition, research about the tumorigenesis mechanism of CIN presents clues about its function during the transformation from normal stem cells to CSCs as certain types of stem cells have been shown to accumulate structural and numerical chromosomal aberrations after long-term culture and is ultimately transformed to CSCs 16, 17. A special group of cells that can self-renew, proliferate infinitely, and exhibit multilineage differentiation potential, CSCs are identified as the source of tumorigenesis, progression, and poor treatment outcome in malignance 18, 19. Moreover, these properties aresummarized as “stemness.” These CSCs share similar levels of potency to produce cell populations with varying degrees of differentiation, which not only sustains the growth of tumors but also generates high heterogeneity in them. This special group of cells in tumors are also characterized by similar cell markers with normal stem cells, such as SOX2, NANOG, and Oct4. Canonical signaling pathways regulating stemness also play a crucial part in CSCs 19, 20.

In this study, we aimed to explore the unknown molecular mechanisms allowing SPC25 to mediate hepatocarcinogenesis, as well as its distinct clinical features in diverse sample sets of HCC. We detected the SPC25 expression in our clinical samples and focused on its clinical relevance and verified the result using mRNA sequencing data from The Cancer Genome Atlas (TCGA) database for HCC. Through an *in vitro* experiment, we found that SPC25 overexpression could facilitate the proliferation, migration, and invasion of HCC cells, together with resistance to chemotherapeutic drugs. Notably, SPC25 overexpression also promoted the sphere formation ability of HCC cells. In animal models, SPC25 accelerated the growth of HCC xenograft tumors. Mechanistically, SPC25 activated the DNA-PK-Akt-NICD signaling cascade in HCC cells to regulate the stemness of HCC. Our study indicated that SPC25 was a potential prognostic factor, and its biological function was performed by the DNA-PK-Akt-NICD pathway in HCC.

## Materials and methods

### Patients and samples

A total of 37 fresh HCC tissue specimens paired with nontumorous liver tissues for real-time quantitative reverse transcription-polymerase chain reaction (RT-PCR) were extracted from primary HCC patients who underwent hepatectomy in Sun Yat-sen University Cancer Center (Guangzhou, China) from 2005 and 2008. After resection, fresh tissues were immersed immediately in RNAlater® (Ambion, Austin, TX, United States), stored overnight at 4 °C and then at -80 °C until RNA isolation. RT-PCR analysis was then conducted. For immunohistochemistry (IHC), 142 HCC tissues were collected from Sun Yat-sen University Cancer Center from 2005 and 2008. The use of human samples was approved by the Institutional Review Board of SYSUCC (Approval No. GZR2019-287).The requirement for informed consent was waived by the institutional review committee. All experiments involving humans were carried out in accordance with the Code of Ethics of the World Medical Association (Declaration of Helsinki).

### Cell lines

HCC cell lines (HepG2, Hep3B, HuH-7, and SK-Hep-1) were purchased from the Cell Bank of the Chinese Academy of Sciences (Shanghai, China). They were cultured in Dulbecco's modified Eagle's medium (Gibco, Thermo Fisher Scientific Inc., Waltham, MA, United States) supplemented with 10% fetal bovine serum (FBS; Gibco, Thermo Fisher Scientific Inc.) and then maintained in a humidified atmosphere of 5% CO2 at 37 °C.

### Lentiviral vector construction and cell infection

Endogenous SPC25 expression was downregulated using two SPC25 small interfering RNAs (siRNAs)—siSPC25-1 (5′-GGACTAAGAGATACCTACA-3′) and siSPC25-2 (5′-CTGCAGATTGTATAAAGA-3′). The siRNAs were transfected with Lipofectamine™ RNAiMAX Transfection Reagent (Thermo Fisher Scientific Inc.), following the manufacturer protocol. For the stable overexpression or knockdown of SPC25 expression in HCC cells, recombinant lentiviruses carrying a human SPC25 overexpression plasmid, short hairpin RNA (shRNA), or corresponding empty vectors were purchased from OBiO Technology Corp. (Shanghai). The shRNA target sequences shSPC25-1 (5′-GGACTAAGAGATACCTACA-3′), and shSPC25 (5′- CTGCAGATTGTATAAAGA-3′) were used. Target cells were infected with lentiviruses for 24 h in accordance with manufacturer instructions, followed by selection with 2 μg/mL of puromycin after 48 h. The efficiency of overexpression and knockdown was verified by qPCR and Western blot analysis 7 days after the selection. The stable infection cell lines were designated as Huh7-vector, Huh7-SPC25, Hep3B-vector, Hep3B-SPC25, HepG2-shCtrl, HepG2-shSPC25-1, HepG2-sh SPC25-2, SK-Hep1- shCtrl, SK-Hep1-shSPC25-1, SK-Hep1-shSPC25-2.

### Colony formation

A total of 500 cells per well were seeded in 6-well plates and then incubated for 14 d at 37 °C in a humidified chamber with 5% CO2. Cell colonies were subsequently fixed with 4% paraformaldehyde for 15 min and then stained with 1% crystal violet for 15 min. The stained colonies were then counted.

### Cell counting kit-8 cell proliferation assay

Cells were seeded in 96-well plates at a density of 1 × 10^3^ cells/well (100 μL/well). At the testing time point, 10 μL of CCK-8 solution (Dojindo Laboratories, Kumamoto, Japan) was added to each experimental well and then incubated for 2 h at 37 °C. The absorbance was measured at 450 nm with an automatic microplate reader (Molecular Devices, Sunnyvale, CA).

### Apoptosis assay

Following transfection for 48 h, cells were harvested and washed with phosphate‑buffered saline (PBS) three times and then resuspend cells in Annexin V Binding Buffer at a concentration of 0.25-1.0 x 10 cells/mL. Add 5 µL of APC Annexin V and 5 µL of 7-AAD Viability Staining Solution (Biolegend). Gently vortex the cells and incubate for 15 min at room temperature (25°C) in the dark. Add 400 µL of Annexin V Binding Buffer to each tube. Analyze by a flow cytometer.

### Spheroid formation assay

Cells indicating treatment (1.2×10^4^ cells/well) were seeded in the Ultralow Attachment 6-well Plate (Corning Inc., Corning, NY, United States) and then cultured in serum-free F12/K Medium (Gibco, Carlsbad, CA, United States), supplemented with the commercial hormone mix B27 (Gibco), 20 ng/mL EGF (Sigma-Aldrich, St. Louis, MO, United States), 10 ng/ml bFGF, and 1×B27 (Invitrogen). The plate was incubated at 37 °C with 5% CO2 for 7 d without being touched. Tumor spheres measuring 50 μm or larger were counted.

### Transwell migration assay

HCC cells were resuspended and counted after digestion by trypsin with 0.25% EDTA. Subsequently, 5.0 × 10^4^ cells with 200 μL of serum-free medium were placed in the upper compartment of a Transwell chamber (Corning; 24-well insert, pore size: 8 μm). The lower chamber was filled with 10% FBS as a chemoattractant and then incubated for 48 h for the migration assay. After 48 h, the cells on the upper surface of the membrane cells were carefully wiped off with cotton swabs. The cells on the lower surface were fixed, stained with 0.1% crystal violet, and washed with phosphate-buffered saline (PBS). The number of stained cells from five visual fields of each insert were randomly chosen and counted under a light microscope. The experiments were performed in triplicate.

### Invasion assay

The invasion assay was conducted using a Matrigel invasion chamber (BD Bioscience) in a 24-well cell culture plate, following the instructions provided by the manufacturer. Cells with 300 μL serum-free medium were seeded into chamber inserts containing a membrane with 8 μm pores by using the Matrigel Matrix Thin Layer. The bottom of the well was filled with 600 μL FBS. The cells that invaded the lower surface of the membrane were fixed after 48 h, and the cells in the upper chamber were removed. The invaded cells were stained with 0.1% crystal violet. The total number of invaded cells was then determined for 5 independent fields under a light microscope.

### Cell Viability Assay

Cell viability was determined using the CCK-8 cell viability assay. Cells (2×103 cells per well) were seeded in flat-bottomed 96-well plates. After incubation for 24 h, the cells were treated with 5-fluorouracil (5-FU) or cisplatin at varying concentrations. After further incubation for 48 h, 100 μL CCK-8 solution was added to each well, followed by incubation for 2 h at 37 °C. Absorbance at 450 nm was measured using an automatic microplate reader. Relative cell viability was calculated as a percentage of untreated controls. Drug sensitivity was determined from three separate experiments and expressed as the drug concentration required to inhibit proliferation by 50% (IC50). Standard curve-fitting routines (GraphPad Software, Inc., San Diego, CA) were used in the analysis.

### Quantitative real-time polymerase chain reaction

Total RNA was extracted from the 37 paired clinical tissue samples and 5 cell lines (L-02, HepG2, Hep3B, HuH-7, SK-Hep-1) by using the Tissue RNA Purification Kit (ESscience, Shanghai, China) and the RNA Quick Purification Kit (ESscience), respectively, in accordance with the manufacturer protocol. RNA concentration and purity were measured by the absorbance at 260 nm on the NanoDrop ND-1000 Spectrophotometer (Thermo Fisher Scientific, Inc.). First-strand cDNA was synthesized from 1-2 µg of total RNA by using the Fast Reverse Transcription Kit (ESscience) as specified in the manufacturer recommendations. Gene mRNA expression was measured by qPCR using the GoTaq qPCR Master Mix (Promega Corporation, Madison, WI, United States). GAPDH was used as an endogenous control for normalization. The sequences of the primers are listed in Table S1.

### Western blot

Cells for Western blot analysis were washed with PBS and then lysed on ice with radioimmunoprecipitation assay (RIPA) buffer (MilliporeSigma, Burlington, MA, United States) to which protease inhibitor cocktail (MilliporeSigma, Burlington, MA, United States) was added. Fresh human tissue samples were ground to a powder in liquid nitrogen and then lysed with ice-cold RIPA buffer. The cell or tissue lysates were analyzed on 10% gels by standard sodium dodecyl sulfate/polyacrylamide gel electrophoresis and transferred to polyvinylidene fluoride membranes (MilliporeSigma, Burlington, MA, United States). The membranes were blocked with 5% skim milk, and protein bands were reacted with the appropriate primary antibodies by incubating at 4 °C overnight. After the membranes were washed three times by using Tris-HCl buffered saline with 0.1% Tween-20, the protein bands were reacted with horseradish peroxidase (HRP)-conjugated secondary antibody. The immunoreacted protein bands were subsequently visualized using an enhanced chemiluminescence detection system (Bio-Rad Laboratories, Hercules, CA, United States). The primary antibodies used in this experiment are listed in Table S2.

### Human Stem Cell Array

HCC cells transfected with indicated plasmids were harvested after the confirmation of the SPC25 expression level. Cell lysates were analyzed with a Human Stem Cell Array C1 (RayBiotech) following manufacture instructions.

### Chromatin immunoprecipitation assay

The chromatin immunoprecipitation (ChIP) assay was performed in accordance with the manufacturer instructions (Cell Signaling Technology). Anti- RBP-Jκ (Cell Signaling Technology) and anti-NICD (Cell Signaling Technology) antibodies were used to immunoprecipitate the chromatin in HCC cells. RT-PCR was performed using primers identified for the RBP-Jκ binding site in the SOX2 and NANOG promoter region as follows: 5′- GGTTCCCAAGAA -3′, 5′- TCTTCCCATCAT -3′.

### Immunocytochemistry staining

Cells with different treatments were fixed with 4% paraformaldehyde at room temperature for 20 min, washed with 1X PBS, permeabilized with 0.1% Triton X-100 (#28314, Thermo Fisher Scientific Inc., Waltham, MA, United States) in 0.01M PBS (pH 7.4) for 10 min, and rinsed three times with PBS for 5 min each. The samples were subsequently blocked with 0.2% bovine serum albumin for 1 h, air-dried, and rehydrated in 1X PBS.

The cells were then incubated with rabbit monoclonal antibody against SOX2 (#14962S, Cell Signaling Technology Inc., Danvers, MA, United States), NANOG (#4893S, Cell Signaling Technology Inc., Danvers, MA, United States) diluted, or γH2AX (C2035S, Beyotime) in 1X PBS containing 3% normal goat serum at room temperature for 1 h. They were then washed thrice with 1X PBS for 5 min each. The cells were incubated with Alexa Fluor® 488 Goat Anti-Rabbit IgG Antibody (Invitrogen) diluted 1:250 in 1X PBS containing 3% normal goat serum at room temperature for 1 h. The cells were subsequently washed 3 times with 1X PBS and incubated with DAPI solution (Solarbio Science & Technology Co., Ltd., Beijing, China) for nuclear staining. Cell images were taken under a confocal fluorescence microscope.

### Immunohistochemistry

Tissue sections were deparaffinized with xylene, rehydrated, subjected to antigen retrieval by high pressure, and incubated with 3% hydrogen peroxide for 10 min. The tissues were then incubated with goat serum for 1 h to block endogenous peroxidase activity and nonspecific staining. Slides were incubated with the appropriate primary antibodies in a humidified chamber at 37 °C for 1 h. After being washed 4 times with PBST (PBS with 1% Tween-20), the slides were incubated with the HRP-conjugated secondary antibody by using the EnVisionTM Detection Kit (Gene Tech Co., Ltd., Shanghai. China) for 30 min and were then stained with 3, 3′-diaminobenzidine tetrahydrochloride. The sections were ultimately counterstained with hematoxylin, followed by dehydration, clearance, and evaluation.

Staining intensity was determined by multiplying the staining value (0, negative; 1, low; 2, medium; and 3, high) and the extent of stained cells (0, 0%; 1, 1%-25%; 2, 26%-75%; and 3, 75%-100%). Five random fields per sample were assessed using a light microscope (Olympus, Tokyo, Japan). The final scores were calculated by multiplying the scores of the intensity by the scores of the extent. All samples were divided into four grades: 0, -; 1-3, +; 4-6, ++; and 7-9, +++. Values with “-” or “+” were grouped into “low expression”, whereas those with “++” or “+++” were grouped into “high expression.” The scores were calculated independently by two experienced pathologists who were blinded to the clinical outcomes.

### Animal models

Female immunodeficient NOD-Prkdc(em26cd52)Il2rg(em26Cd22)/Nju (NCG) mice aged 4 weeks were purchased from GemPharmatech Co., Ltd. (Nanjing, China). All animal experiments were approved by the Institutional Animal Care and Use Committee of the Sun Yat-sen University Cancer Center (SYSUCC) (Approval No. L102012019010E). All cells used in the animal study were stably expressed luciferase. The mice were subcutaneously injected with a total of 3.5×10^6^ tumor cells suspended in 100 μL of PBS containing 50% Matrigel Basement Membrane Matrix (BD Biosciences) at the right axilla and then randomly assigned to 4 groups, each consisting of 5 mice. The tumor volume was measured once per week using an *in vivo* imaging system (PerkinElmer, IVIS Lumina Series III, United States) on Days 7, 14, and 21. All xenografts were harvested, photographed, weighed, and embedded in paraffin on Day 22. IHC staining was performed to measure the expression levels of SPC25, Ki67, SOX2, and NANOG.

### Statistical analysis

Data were presented as mean ± standard deviation (SD) by using Student's t-test or the χ^2^ test. Survival analysis was conducted using the Kaplan-Meier method. The χ^2^ test was performed to analyze the relationship between SPC25 expression and the clinicopathological characteristics. Based on the variables selected via univariate analysis, the multivariate Cox proportional hazards model was used to determine the independent prognostic factors for HCC. The association between SPC25 and SOX2, or NANOG expression in HCC tissues was calculated using the Pearson correlation test. The differences between groups were evaluated using Student's two-tailed t-test and one-way ANOVA. P<0.05 was considered statistically significant. The statistical analysis and figure generation were performed using the Prism 8.0 software (GraphPad Software, Inc., San Diego, CA, United States) and SPSS version 25.0 (IBM Corporation, Armonk, NY, United States).

## Results

### SPC25 is highly expressed in HCC and related to poor survival in patients

**Figure 1:**
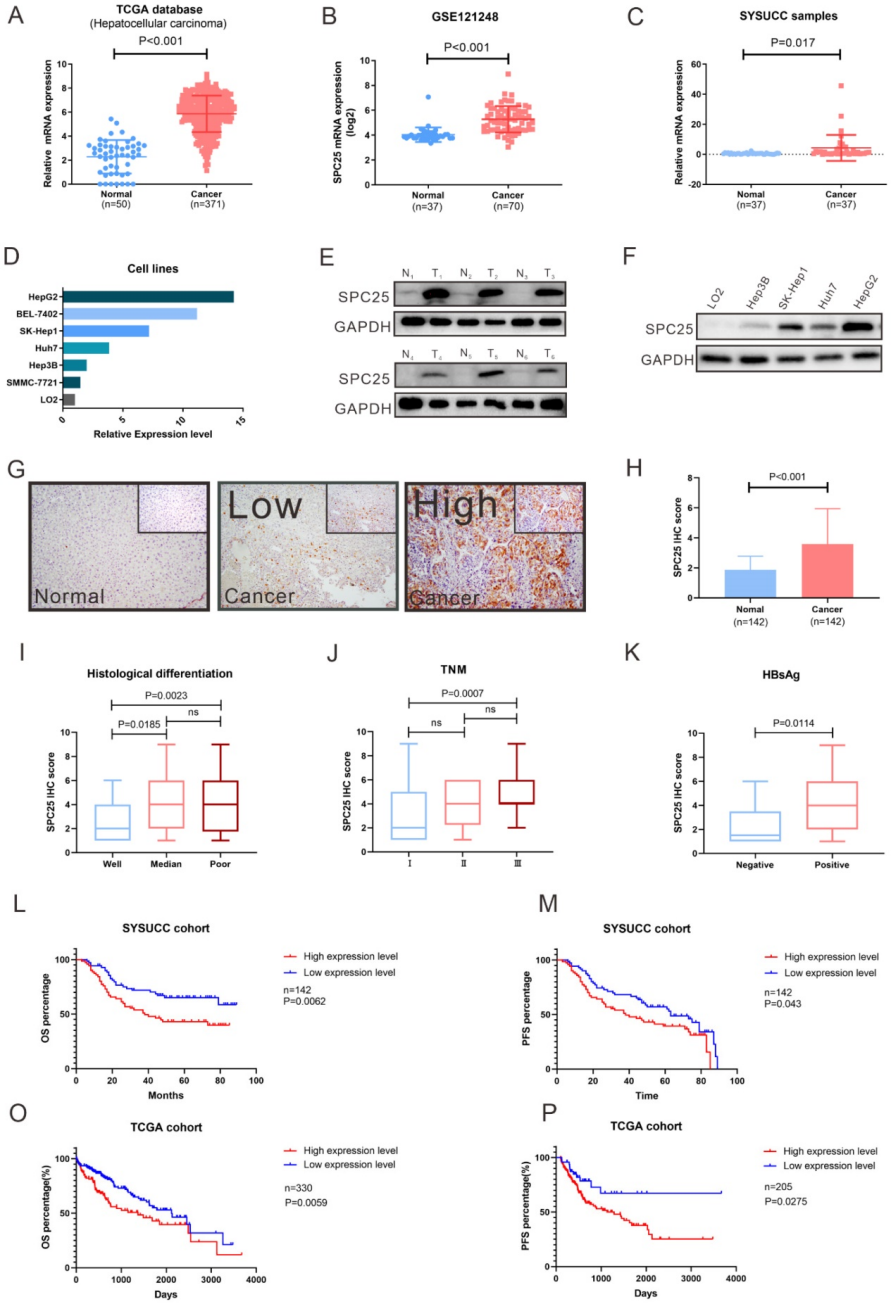
** SPC25 is upregulated in tumor tissues and is significantly associated with poor survival in HCC.** (**A-D**) SPC25 mRNA expression levels in the liver hepatocellular carcinoma (LIHC) dataset from the TCGA database (A) and the public GEO liver cancer dataset GSE121248. (B) 37 pairs of cancer tissues (C) and adjacent normal liver tissues from SYSUCC samples and several HCC cell lines (SMMC7721, Hep3B, Huh7, SK-Hep1, and BEL-7402) and human normal hepatocytes (LO2) (D). (**E-F**) Representative images of Western blot analysis conducted on 37 pairs of HCC tumor tissues (T) and adjacent normal liver (N) tissues (E), and in several HCC cell lines (SMMC7721, Hep3B, Huh7, SK-Hep1, and BEL-7402) and human normal hepatocytes (LO2) (F). (**G-H**) Representative images of the immunocytochemistry staining of adjacent normal liver tissues, tumor tissues with low SPC25 expression, and tumor tissues with high SPC25 expression, and statistical analysis of the scores based on staining intensity (G) and range and statistical analysis of the score based on the staining intensity and range (H). (**I-K**) Expression status of SPC25, classified by histological differentiation (I), TNM (J), and HBsAg expression (K). (**L-M**) Kaplan-Meier analysis of OS (L) and PFS (M) on the basis of the SPC25 expression status in 142 HCC patients from the SYSUCC cohort. (**O-P**) Kaplan-Meier analysis of OS (O) and PFS (P) on the basis of the SPC25 expression status in 330 HCC patients from the TCGA cohort.

To determine the roles of SPC25 in HCC progression, we first analyzed the SPC25 expression in liver cancer tissues from the TCGA and Gene Expression Omnibus (GEO) datasets (GSE121248). The data reflected an increase in the SPC25 mRNA expression in 371 HCC samples relative to 50 adjacent normal samples (Fig. 1A-1B). We then examined the SPC25 expression in our clinical samples by conducting qRT-PCR, Western blot, and IHC separately. Consistently, the mRNA expression level of SPC25 was significantly upregulated in 37 paired HCC samples relative to the adjacent nontumorous tissues (Fig. 1C) and was also upregulated in HCC cell lines (HepG2, BEL7402, SK-Hep1, Huh7, Hep3B and SMMC-7721) compared to normal liver cell line (LO2) (Fig. 1D). Western blot analysis showed that SPC25 protein expression was also elevated in six pairs of fresh HCC specimens compared to their adjacent normal liver tissues (Fig. 1E) and HCC cell lines (HepG2, SK-Hep1, Huh7 and Hep3B) (Fig. 1F). The IHC results calculated by the score of staining intensity and scope in the SYSUCC cohort with 142 HCC samples demonstrated that SPC25 was highly expressed in the cancer region (“Cancer” in Fig. 1G-1H) but poorly expressed — or even not at all — expressed in the adjacent normal region (“Normal” in Fig. 1G-1H). Among the clinical characteristics of these samples, SPC25 expression was upregulated in patients with poor histological differentiation (Fig. 1I), advanced TNM (T: tumor, N: lymph nodes, M: metastasis) stage (Fig. 1J), and HBV infection (Fig. 1K), and the correlation analysis of these characteristics with SPC25 was also showed in Table 1.

For the prognostic value of SPC25, we explored the overall survival (OS) and progression-free survival (PFS) among the patients from the SYSUCC and TCGA cohorts respectively. SPC25 expression was significantly associated with the survival of patients with HCC regardless of the patient cohort (i.e., SYSUCC or TCGA cohort). The patients with high SPC25 expression showed shorter OS and PFS, whereas those with low SPC25 expression demonstrated longer OS and PFS (Fig. 1O-P). In our SYSUCC cohort, the median OS in high SPC25 and low SPC25 groups were 38.0 and 49.0 months, and the median lengths of PFS were 38.0 and 63.0 months. In addition, univariate and multivariate Cox regression analysis suggested that SPC25 was an independent prognostic factor for OS in HCC (Table 2). In conclusion, the results of the expression analysis suggested that SPC25 was a promising factor related to the development and prognosis of HCC.

### SPC25 promotes cell proliferation in hepatocellular carcinoma

**Figure 2:**
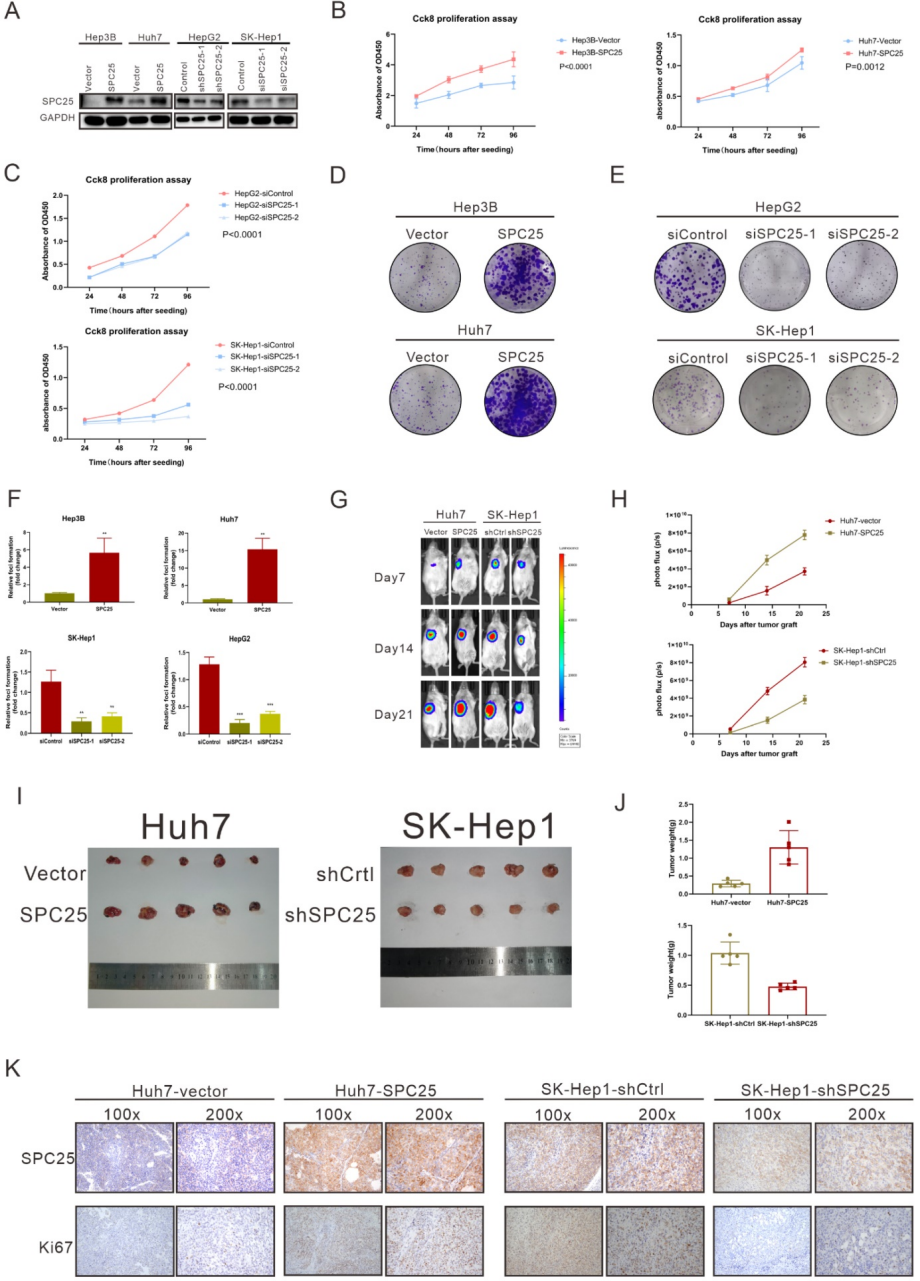
** SPC25 promotes hepatocellular carcinoma cell proliferation *in vitro and in vivo*.** (**A**) Expression verification of HCC cell lines transfected with lentiviruses carrying a human SPC25 overexpression plasmid 7 days after the selection by puromycin and 72 hours after the transfection with SPC25 small-interfering RNAs. (**B-C**) CCK-8 assay and (**D**-**F**) colony formation assay showing the cell growth of the indicated HCC cells. (**G**-**H**) Representative images (**G**) and photo flux (**H**) of the tumor growth of Huh7-vector, Huh7-SPC25, SK-Hep1-shControl, and SK-Hep1-shSPC25 in NOD-SCID mice on Days 7, 14, and 21. (**I**) Images of the dissected tumors from the nude mice. (**J**) Tumor weight of each group. (**K**) Representative images of IHC staining with anti-SPC25 and anti-Ki67 on the tumors of the tested mice. Images at 100× and 200× magnification. All *in vitro* experiments in triplicate. Error bars represent the mean ± SD. *P < 0.05; **P < 0.01; *** P < 0.001; ****P < 0.0001; ns means non-significant.

To verify the ability of SPC25 to promote tumorigenesis *in vitro* and *in vivo*, we selected several HCC cell lines to explore its biological function. Hep3B and Huh7, which poorly expressed SPC25, were transfected with recombinant lentivirus carrying a human SPC25 overexpression plasmid. Meanwhile, the highly expressing cell lines HepG2 and SK-Hep1 were transfected with SPC25 siRNAs for *in vitro* experiments and the lentivirus carrying target shRNA for *in vivo* experiments. SPC25 expression in these stable cell lines was also detected by Western blot analysis after 7-days selection by puromycin in HCC cells transfected with recombinant lentivirus and in HCC cells at the time of 72 hours after transfecting with SPC25 siRNAs (Fig. 2A). After the certification of expression level of SPC25 in the indicated HCC cells, we performed a series of experiments *in vitro* and *in vivo* to verify the difference of proliferating ability between these HCC cells with diverse expression level of SPC25. In foci formation and CCK8 proliferation assays, the cells with overexpressed SPC25 showed increased colonies and proliferation speed, and the cells with the silence of SPC25 demonstrated the reverse effect (Fig. 2B-2F). However, the apoptosis assay analyzed by flow cytometry using the Annexin V-APC/7-AAD Apoptosis kits showed no difference in SPC25 overexpression or knockdown from those of their control groups (Fig. S1). Apart from the experiments performed in HCC cell lines, we further explored the pro-proliferating ability of SPC25 in normal hepatic cell lines. As was shown in Fig. S2A-S2B, LO2 cells with overexpressed SPC25 also showed increased proliferation speed and formed more cell colonies, which further verified that the overexpression of SPC25 could not only promoted the proliferation of HCC cells but also normal hepatic cells.

A subcutaneous tumor formation assay was used *in vivo* to investigate the effect of SPC25 on tumor growth (n=5/group). The tumors grew faster in mice transplanted with Huh7-SPC25 in the Huh7 transplanted with an empty vector (Huh7-vector). However, the tumors in mice transplanted with SK-Hep1-shSPC25 had a smaller volume than in those transplanted with SK-Hep1-shCtrl (Fig. 2G-2J). After harvesting all xenograft tumors, we further performed IHC analysis *in situ* for these subcutaneous tumor samples. The cell proliferation index of tumors was evaluated by Ki67 staining. The staining intensity of Ki67 was found to be markedly reduced in SK-Hep1-shSPC25 tumors; moreover, the intensities were higher in Huh7-SPC25 tumors than in its respective control groups (Fig. 2K). In summary, the upregulation of SPC25 could have promoted the proliferation of HCC cells.

### SPC25 induces the cancer stem-like cell phenotype of HCC cells

**Figure 3:**
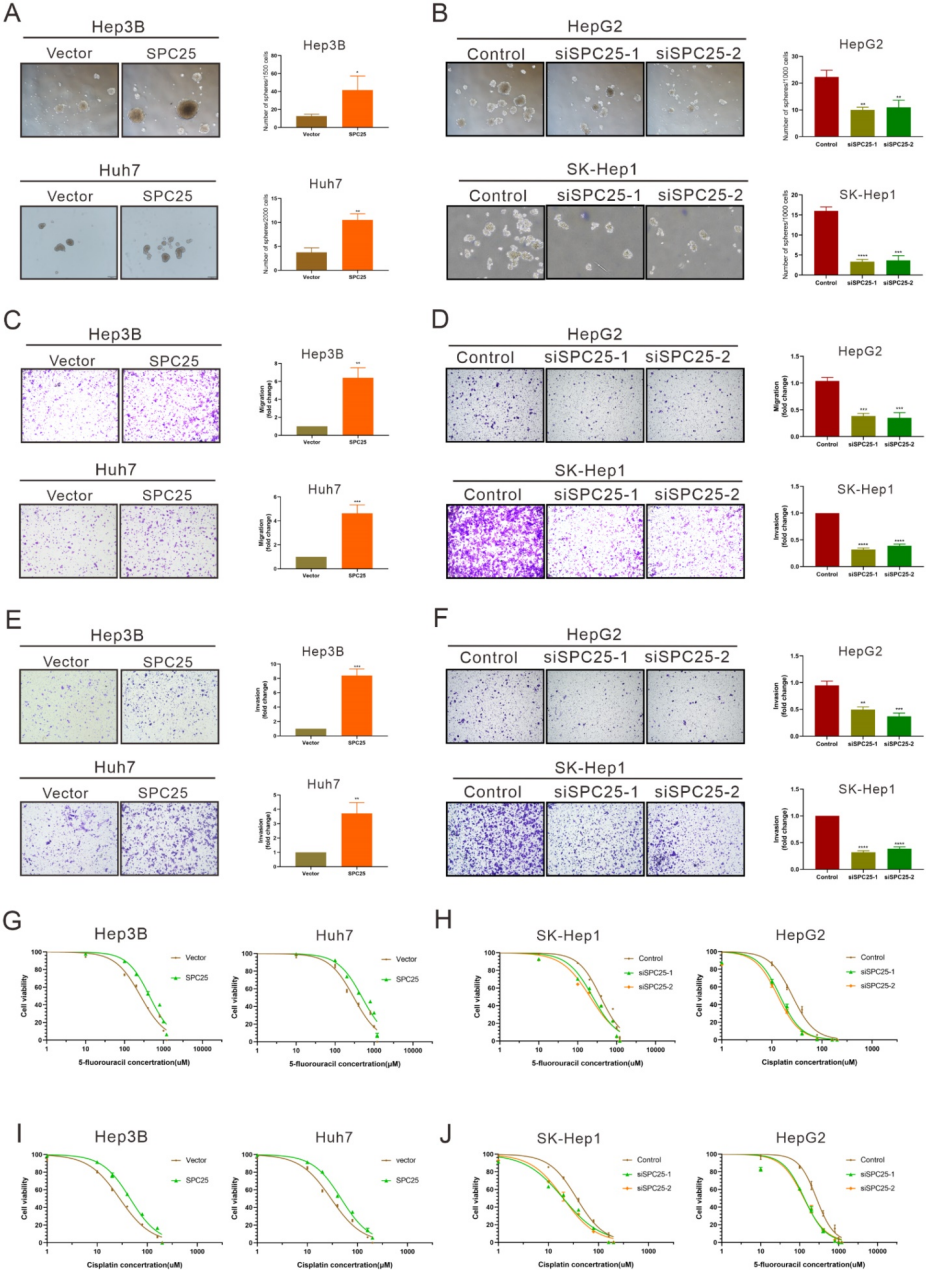
** SPC25 induces the cancer stem-like cell phenotype of HCC cells.** (**A-B**) Spheroid formation assay demonstrating the size and number formed by indicated HCC cells cultivated in spheroid formation medium for 7 days. (**C-D**) Transwell assay showing the number of HCC cells migrating from the upper chamber to the bottom well during 24 hours. (**E-F**) Invasion assay indicating the number of HCC cells migrating from the upper chamber, which was coated with Matrigel to the bottom well during 24 hours. (**G-J**) Huh7, Hep3B, and SK-Hep1 cells transfected with the indicated constructs, treated with 5-FU (G-H) and cisplatin (I-J) at different concentrations for 24 h; cell viability measured using the CCK-8 assay and demonstrated byconcentration-response curves. Error bars represent the mean ± SD. **P < 0.01; ***P < 0.001; ****P < 0.0001.

Previous studies have suggested the function of SPC25 in promoting the stemness of cancer cells 7, 8. Thus, we further performed a series of related biological experiments on stemness. The spheroid formation assay first showed that the cells with SPC25 overexpression formed more and larger spheroids, whereas the cells transfected with siRNAs of SPC25 showed the opposite results (Fig. 3A-3B). Biologically, the property prone to metastasis is associated with the properties of stem cells. The link between metastasis and CSCs has recently been studied in various types of cancer. Cancer cells that exhibit epithelial-to-mesenchymal transition (EMT) and increased metastatic phenotype 21. Therefore, the effect of SPC25 on the migration and invasion of HCC cells was verified. Transwell assays demonstrated that SPC25 overexpression increased the migration of cells from the upper chamber to the bottom well in Hep3B-SPC25 and Huh7-SPC25 cells relative to those in Hep3B-vector and Huh7-vector, respectively. SPC25 silenced and inhibited cell movement in SK-Hep1-siSPC25 and HepG2-siSPC25 cells (Fig. 3C-3D). Meanwhile, invasion assays showed that SPC25 enhanced the ability of cell invasion in HCC cells (Fig. 3E) and impaired cell invasion with SPC25 silencing (Fig. 3F). These occurrences indicated that SPC25 promoted the migration ability of HCC cells. However, when we performed the spheroid formation assay, migration and invasion assay in normal hepatic cells transfected with indicated plasmids, we found that the overexpression of SPC25 could only promoted the migration and invasion of LO2 cells but was insufficient to promote the spheroid formation in LO2-SPC25 cells (Fig. S2C-2E). Given the challenges of demonstrating the functional enhance on stemness and migration, we could not provide the related *in vivo* data, which was the limitation for this part of our study. Chemotherapy resistance is another property of CSLCs 22. Thus, we assessed cell viability in HCC cells treated with 5-FU and cisplatin. The results showed that Hep3B-SPC25 and Huh7-SPC25 cells were less sensitive to 5-FU and cisplatin than their repective control groups. In the 5-FU assay, the IC50 values of Hep3B-SPC25 and Huh7-SPC25 were 437.8 and 497 μM, respectively, whereas those of Hep3B-vector and Huh7-vector were 258.3 and 304.3 μM. As for the assessment for cisplatin, the IC50 values of Hep3B-SPC25 and Huh7-SPC25 were 45.72 and 47.40 μM, respectively, whereas those of Hep3B-vector and Huh7-vector were 28.27 and 29.85 μM. Consistent with the cell viability assay in HCC cells with the silence of SPC25 expression, the sensitivities to 5-FU and cisplatin of SK-Hep1-siSPC25 and HepG2-siSPC25 cells were also promoted (Fig. 3G-3J).

On the basis of the aforementioned results, SPC25 induced the typical CSLC phenotypes of HCC cells and facilitated HCC progression and metastasis.

### SPC25 upregulates the expression of stemness-related markers

**Figure 4:**
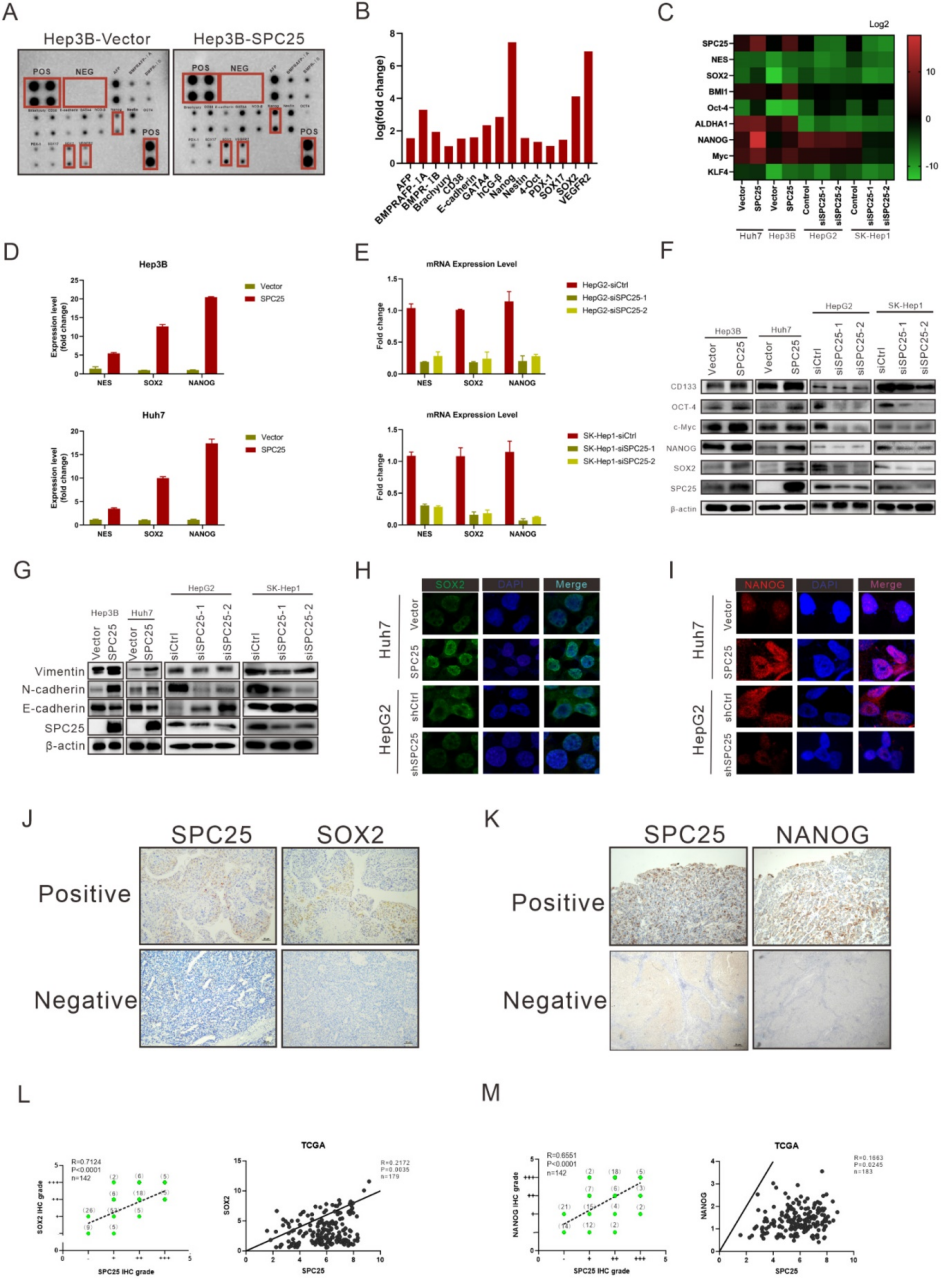
** SPC25 upregulates the expression of stemness-related markers.** (**A**-**E**) Human stem cell proteome array (A-B) (POS: Positive Control Spots; NEG: Negative Control Spots) and PCR array (C-E) for stemness typical markers in indicated HCC cell lines. (**F-G**) Western blot analysis of the expression of several stemness markers (CD133, OCT4, c-Myc, NANOG, and SOX2) (F) and EMT-related genes (vimentin, N-cadherin, and E-cadherin) (G) in Hep3B-vector, Hep3B-SPC25, Huh7-vector, Huh7-SPC25, HepG2-siControl, HepG2-siSPC25-1, and HepG2-siSPC25-2 cells. (**H-I**) IF staining for the cell colonization of Sox2 (H) and Nanog (I) in Huh7-vector, Huh7-SPC25, HepG2-siControl, HepG2-siSPC25-1, and HepG2-siSPC25-2 cells. (**J**) Representative images of the IHC staining showing the correlation between SPC25 expression and Sox2 expression (**K**) Representative images of IHC staining showing the correlation between SPC25 expression and Nanog expression. (**L**) Correlation analysis demonstrating the association between the IHC grades of Sox2 and SPC25 in 142 HCC patients and the relative expression levels of Sox2 and SPC25 on the TCGA database. (**M**) Correlation analysis demonstrating the association between the IHC grades of Nanog and SPC25 in 142 HCC patients and the relative expression levels of Nanog and SPC25 on the TCGA database. Error bars represent the mean ± SD. *P < 0.05; **P < 0.01; *** P < 0.001; ****P < 0.0001; ns means non-significant.

We determined the correlation between SPC25 and cell stemness from the aforementioned biological assays. This association was further explored at the molecular level. We first performed the human stem cell proteome array by cell lysis from Hep3B-vector and Hep3B-SPC25 to screen differentially expressed molecules related to stemness. The results indicated that SPC25 overexpression upregulated a series of typical stemness-associated molecules. The most significantly upregulated molecules among them were Nanog, Vegfr2, and Sox2 (Fig. 4A-B). Accordingly, we examined the transcription level of these stemness-associated molecules by qRT-PCR. Consistent with the results of protein array analysis, the expression level of a group of stem-associated molecules was increased in Hep3B-SPC25 and Huh7-SPC25 cells but decreased in HepG2-siSPC25 cells and SK-Hep1-siSPC25 cells relative to those in their control cells, respectively. The mRNA level results showed that NANOG, SOX2, and NES were the top three differentially expressed genes (Fig. 4C-4E). Further, the Western blot analysis results showed that the typical stemness CD133, Oct4, c-Myc, Nanog, and Sox2, were upregulated in Hep3B-SPC25 and Huh7-SPC25 cells relative to those in the control groups of the cell lines. These markers were downregulated in HepG2-siSPC25 cells and SK-Hep1-siSPC25 cells (Figure 4F). With regard to our experiment on metastasis, the expression of vimentin and N-cadherin was increased, whereas that of E-cadherin was inhibited in Hep3B-SPC25 and Huh7-SPC25 cells (Figure 4G). This aspect of the results further implied the involvement of SPC25 in EMT regulation, which was also one of the features of CSLCs.

Given these data, we chose the most significantly expressed markers, Sox2 and Nanog, to analyze their cell colocalization by immunofluorescence assay. The results demonstrated that nuclear Sox2 and Nanog were parallelly expressed with SPC25 in HCC cells, which was markedly increased in SPC25 overexpression cells and decreased in SPC25 knockdown cells (Fig. 4H-4I). We then detected the expression of Sox2 and Nanog in the samples collected from clinical HCC patients and xenograft tumors from immunodeficient NCG mice by IHC staining. Sox2 and Nanog were acknowledged as stemness markers and were poorly expressed in most HCC patients; however, they were markedly upregulated in tissues with highly expressed SPC25 (Fig. 4J-4K). After their expression scores were calculated, statistical analysis indicated that the expression levels of Sox2 and Nanog exhibited a high positive correlation with SPC25. The similarly positive correlation was displayed based on the gene expression data collected from the TCGA database (Fig.4L-4M). With regard to the assessment of xenograft tumors, IHC staining also demonstrated that the tumors formed by Huh7-SPC25 exhibited increases in intensity in SOX2 and NANOG, together with a decrease in intensity in tumors formed by SK-hep1-shSPC25 (Fig. S2).

In summary, we verified the correlation between SPC25 and stemness from different aspects, including mRNA expression, protein expression, cell localization, clinical samples, and animal model. All results suggested that SPC25 upregulated the expression of stemness-related markers.

### SPC25 upregulation increases the incidence of DNA damage and activates the DNA-PK/AKT/Notch1 signaling pathway in HCC

**Figure 5:**
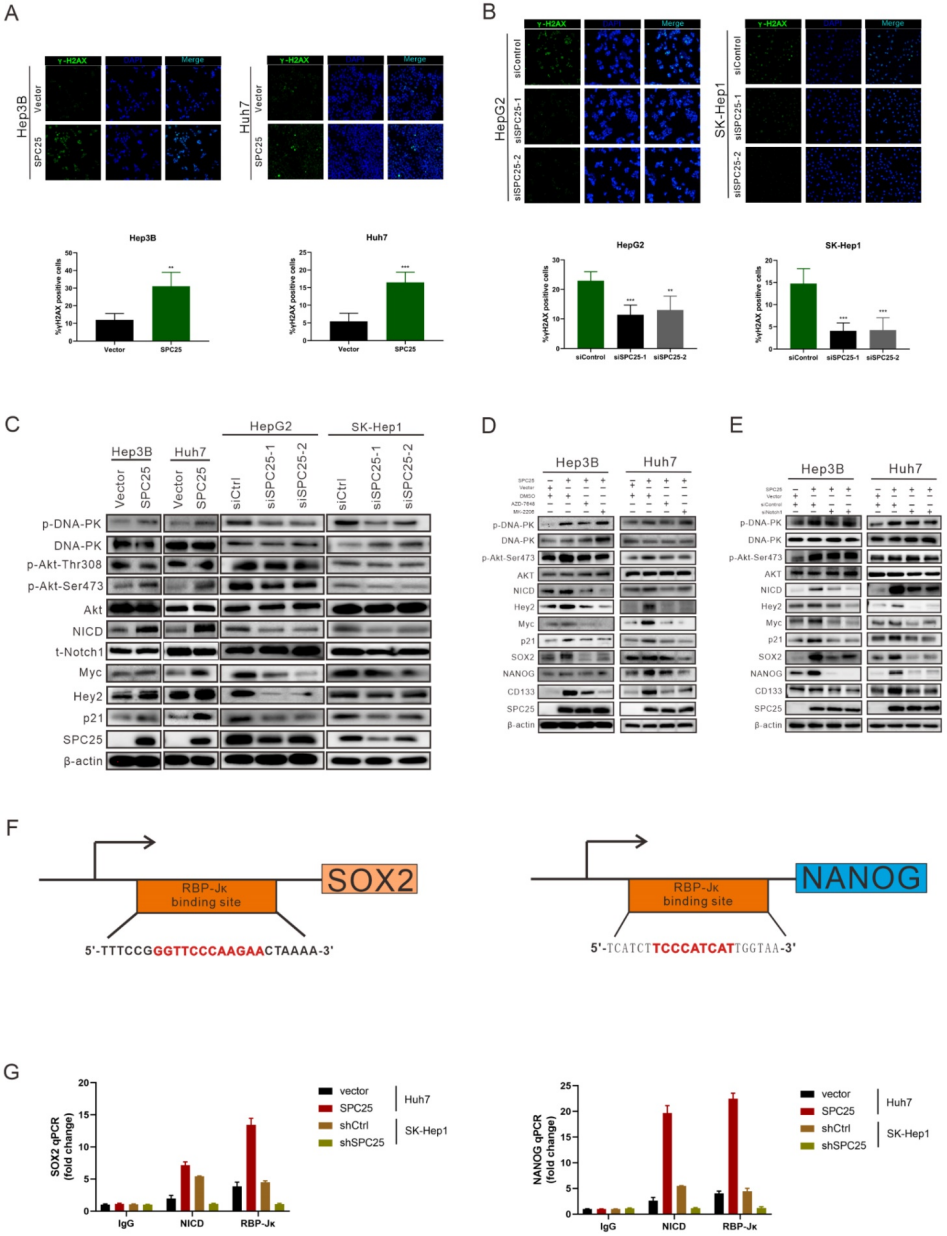
** SPC25 upregulation increases the incidence of DNA damage and activates the PI3KDNA-PK/AKT/Notch1 signaling pathway in HCC. (A-B)** Images of γH2AX immunostaining in indicated HCC cells 72 hours after transfection with different plasmids. **(C)** Western blot analysis of the activation levels of DNA-PK, AKT, and Notch1 signaling pathway in indicated HCC cells. **(D)** Huh7-SPC25 and Hep3B-SPC25 cells were treated with 5 µM AZD-7648 and 5 µM MK-2206 respectively; changes in the activation of the DNA-PK/AKT and Notch1 signaling pathway and the expression levels of SOX2, NANOG, and CD133. **(E)** Huh7-SPC25 and Hep3B-SPC25 cells were transfected with Notch1-specific siRNAs, and changes in the activation of the DNA-PK/AKT and Notch1 signaling pathway and the expression levels of SOX2, NANOG, and CD133. **(F-G)** An RBP-Jκ binding site, as determined by the analysis of the SOX2 and NANOG promoters. CHIP was performed using IgG, NICD, and RBP-Jκ antibodies, followed by quantitative RT-PCR in indicated cells. Error bars represent the mean ± SD. *P < 0.05; **P < 0.01; *** P < 0.001; ****P < 0.0001; ns means non-significant.

According to the former researches, the abnormal production of any of the NDC80 complex genes can cause chromosomal aberration and instability of the genome 23. Therefore, we performed the detection of DNA damage by γ-H2AX staining in indicated HCC cells. As it was shown in Fig. 5A, the percentage of γ-H2AX positive cells and staining intensity was increased in Hep3B-SPC25 and Huh7-SPC25 cells compared with their control group cells. Additionally, the expression status of γ-H2AX was decreased in SK-Hep1 cells and HepG2 cells with the silence of SPC25 expression (Fig. 5B). These results demonstrated that SPC25 upregulation increases the incidence of DNA damage.

We subsequently explored the specific signaling pathway mediating the effect of SPC25 on proliferation and stemness. Based on the results of the γ-H2AX expression, we inferred that SPC25 upregulation promoted the occurrence of DNA damage the formation of DNA double-strand break (DSB). Correspondingly, there were plenty of reliable researches uncovering that DNA-dependent protein kinase (DNA-PK), a serine/threonine protein kinase complex composed of a heterodimer of Ku proteins (Ku70/Ku80) and the catalytic subunit DNA-PKcs, is a critical component of the response to DNA damage 24. So we detected the activation of DNA-PK in HCC cells with different treatments. The results of western blot (Fig. 5C) demonstrated that the phosphorylation of DNA-PK at Thr2609 site was significantly increased in Hep3B-SPC25 and Huh7-SPC25 cells but decreased in SK-Hep1-siSPC25 cells and HepG2-siSPC25 cells 72 hours after transfection of indicated plasmids.

Next we aimed to continue exploring the downstream signaling molecules that promoted the cell stemness. Current studies have shown that AKT, Notch1, Wnt/β-catenin, and ERK1/2 are CSC-critical signaling activation pathways to regulate the cell pluripotency and self-renewal. Therefore, the effect of SPC25 on the activation of these signaling pathways was subsequently determined. Western blot analysis showed that only the AKT and Notch1 pathways could be activated significantly by SPC25 overexpression (Fig. S4A). AKT phosphorylation at Ser473 was increased in cells showing SPC25 overexpression but decreased in cells exhibiting SPC25 knockdown, but AKT phosphorylation at Thr308 remain unchanged. With regard to the Notch1 pathway, the expression of activated Notch1 (NICD) and its typical downstream activation markers (p21, Myc and Hey2) was upregulated in HCC cells with SPC25 overexpression and downregulated in HCC cells with SPC25 knockdown (Fig. 5C). To connect the activation of DNA-PK, AKT and Notch1, there were abundant studies verifying that DNA-PKcs activation results in AKT-Ser473 phosphorylation in response to DNA damage 24 and the intertwined relationship between Notch1 and the PI3K/AKT axis was also reported in many researches about the signaling transduction in cancer 25. Therefore, we inferred that the upregulation of SPC25 caused the increased DNA damage and then elevated the phosphorylation of DNA-PK, which sequentially activated AKT and Notch1.

According to previous researches, the activation of the AKT directly promotes the transcription of several stemness-related genes, such as SOX2, NANOG, and OCT4 26. With regard to the Notch1 signaling pathway, the Notch1 receptor is cleaved and then releases the NICD, which migrates into the nucleus and forms a complex with the nuclear proteins of the RBP-Jκ family upon activation. RBP-Jκ acts as a transcriptional activator to activate the expression of target genes after forming a complex with NICD 27. Recent studies have connected AKT and the Notch1 pathway in regulating stemness, and some suggest the presence of a mutually regulated relationship between these two pathways 28, 29.

The next we used specific inhibitors of DNA-PK and AKT to verify their regulative roles in the process that SPC25 upregulation promoted cell proliferation and stemness. We first treated Hep3B-SPC25 and Huh7-SPC25 cells with AZD-7648, a specific inhibitor of DNA-PK that blocks DNA-PK phosphorylation. We then found that DNA-PK activation by SPC25 overexpression was partly blocked as expected 24 hours after the treatment. At same time, the expression levels of the stemness-associated markers that we explored, such as SOX2, NANOG, and CD133, were markedly inhibited by AZD-7648 (Fig. 5D). Notably, the AKT phosphorylation at Ser473 and the expression of several activation markers of Notch1 were also suppressed. On the other hand, we used the specific inhibitor, MK-2206, to inhibit the activation of Akt in Hep3B-SPC25 and Huh7-SPC25 cells, and we found that the expression of the same stemness-associated markers and activation of Notch1 signaling were suppressed along with the decreased phosphorylation of AKT at Ser473 24 hours after the treatment. However, the inhibition had no effect on the activation of DNA-PK in these cells with high expression of SPC25 (Fig. 5D). This occurrence suggests that AKT and Notch1 is potentially the downstream pathway of the DNA-PK in SPC25 overexpression cells.

Furthermore, we silenced Notch1 expression by transfecting Hep3B-SPC25 and Huh7-SPC25 cells with Notch1 siRNA. As shown in Fig. 5E, both AKT and DNA-PK phosphorylation were not affected, but the expression of downstream stemness-associated molecules were decreased significantly 72 hours after the siRNAs of Notch1 transfecting. On the basis of this observation, combined with the previous results revealing the inhibition of the DNA-PK and AKT pathway, we could deduce that SPC25 might regulate the proliferation and stemness of HCC cells by promoting the activation of DNA-PK/Akt pathway and subsequently activating the Notch1 pathway. In addition, we further detected the activation level of PI3K and mTOR, both of which were important signaling molecules connected with AKT, but their unchanged status suggested us that they were not the key components involved in the regulating process of SPC25 upregulation (Fig. 4B).

To further verify that SOX2 and NANOG were the main downstream regulated genes of the DNA-PK/AKT/Notch1 signaling pathway that promoted the stemness of HCC cells with upregulated SPC25 expression, we first used the PROMO website to predicate putative RBP-Jκ binding sites in the SOX2 and NANOG promoter region and identified an RBP-Jκ binding site in the -2007 to -1996 region of SOX2 and in the -2213 to -2202 region of NANOG. ChIP assays were subsequently performed to verify whether the RBP-Jκ-NICD complex was bound to the putative site. As shown in Fig. 5F-5G, RBP-Jκ and NICD were bound directly to the putative RBP-Jκ-binding site in the promoter region in Huh7 cells. These observations indicated an increase in Huh7-SPC25 cells and a significant decrease in SK-Hep1-shSPC25 cells relative to the SK-Hep1-shCtrl cells.

Above experimental results based on the detection of the expression change of several clusters of pivotal proteins in different signaling pathways revealed that the SPC25 overexpression caused increased incidence of DNA damage, which activated the DNA-PK/AKT/Notch1 signaling pathway and subsequently elevated the expression of the critical genes (SOX2 and NANOG) that promoted cell stemness and proliferation.

### Blockade of DNA-PK/AKT/Notch1 signaling inhibits the proliferation and stemness phenotype of HCC cells

**Figure 6:**
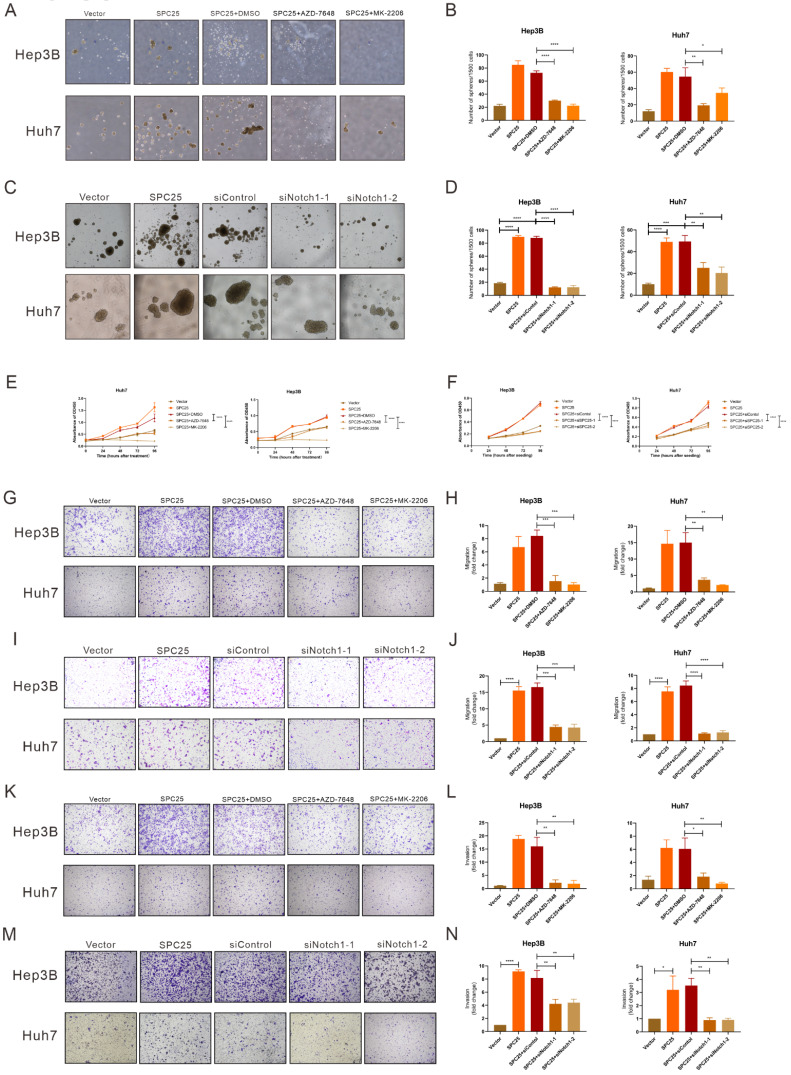
** Blockade of DNA-PK/AKT/Notch1 signaling inhibits the proliferation and stemness phenotype of HCC cells.** (**A**-**B**) Spheroid formation assay demonstrating the size and number formed by Huh7-SPC25 and Hep3B-SPC25 cells treated with 5μM AZD-7648 and 5μM MK-2206 respectively for 7 days. (**C**-**D**) Spheroid formation assay demonstrating the size and number formed by Huh7-SPC25 and Hep3B-SPC25 cells transfected with Notch1-specific siRNAs for 7 days. (**E**-**F**) CCK-8 assay showing the cell growth of Huh7-SPC25 and Hep3B-SPC25 cells treated with 5μM AZD-7648 and 5μM MK-2206 (E) and transfected with Notch1-specific siRNA (F). (**G-J**) Transwell assay showing the indicated number of Huh7-SPC25 and Hep3B-SPC25 cells treated with 5μM AZD-7648 and 5μM MK-2206 (G-H) for 24 hours and 72 hours after the transfection with Notch1-specific siRNAs (I-J). (**K-N**) Invasion assay showing the indicated number of Huh7-SPC25 and Hep3B-SPC25 cells treated with 5 µM AZD-7648 and 5 µM MK-2206 (K-L) for 24 hours and 72 hours after the transfection with Notch1-specific siRNAs (M-N) migrating from the upper Matrigel invasion chamber to the bottom well. Error bars represent the mean ± SD. *P < 0.05; **P < 0.01; *** P < 0.001; ****P < 0.0001; ns means non-significant.

In order to ascertain whether the DNA-PK/AKT/Notch1 signaling pathway mediated the effect of SPC25 on increased proliferation and the stemness phenotype of HCC cells, we continued to use AZD-7648 and MK-2206 in 5 µM in the sphere formation medium to inhibit the DNA-PK and AKT activation of Hep3B-SPC25 and Huh7-SPC25 cells respectively. As it was shown in Fig. 5A-5B, the inhibition of DNA-PK and Akt consistently suppressed spheroid formation in the treatment group. Similarly, in Hep3B-SPC25 and Huh7-SPC25 cells transfected with siRNA targeting Notch1, spheroid formation was also inhibited 72 hours after the transfection (Fig. 5C-5D). The effect on proliferation was similar to that of the spheroid formation assay. Both the AKT phosphorylation inhibition and Notch1 silencing suppressed the increased proliferation of cells with SPC25 overexpression, which was verified using the CCK8 proliferation assay (Fig. 6E-6F). We also performed transwell and invasion assays to examine the effect of the DNA-PK/AKT/Notch1 signaling pathway on the migration and invasion of HCC cells. Hep3B-SPC25 and Huh7-SPC25HCC cells were treated with 5μM with AZD-7648 and MK-2206 for 24 hours. And then these cells with different treatments were harvested and seeded into the upper chamber of the transwell plates. Both migration and invasion abilities were suppressed by the use of inhibition of DNA-PK and Akt (Fig. 6G-6H and Fig. 6K-6L). Consistently, 72 hours after the transfection of siRNA specific to Notch1 in Hep3B-SPC25 and Huh7-SPC25 cells, the migration and invasion abilities were also suppressed (Fig. 6I-6J and Fig. 6M-6N).

The aforementioned findings showed that after blocking the activation of DNA-PK/AKT/Notch1 signaling pathways, the stemness phenotype of HCC cells exhibited significantly suppressed self-renewal, proliferation, migration, and invasion.

## Discussion

Abnormal mitosis is a dominant feature found in various diseases, particularly tumors. Kinetochores are the major factors associated with normal and abnormal cell growth, which functions via their regulation of mitotic chromosome segregation. SPC25 is an indispensable component for assembling the NDC80 complex, which plays a key role in the assembly of the microtubule-binding domain of kinetochores and mediates chromosome alignment with the metaphase plate. NDC80 overexpression leads to the permanent hyperactivation of mitotic control points and an increase in the incidence of liver cancer *in vivo*, as determined from studies using a high-expression NDC80 mouse model and a non-transgenic murine model 30, 31. Interacting with SPC24 32, SPC25 mediates chromosome alignment and spindle formation, which are essential for chromosome segregation and functions as an essential component auto-inhibiting the interaction between microtubule and the Ndc80 complex. Therefore, SPC25 can regulate chromosome segregation during cell mitosis, which is a crucial process associated with tumorigenesis 5, 30, 33.

Researches on SPC25 thus far focus on its function in cell mitosis, and its dysregulation has been confirmed to result in abnormal cell division. Data on the correlation between SPC25 and cancerogenesis remain limited. Statistical analysis based on online databases such as TCGA and International Cancer Genome Consortium (ICGC) 7, 10, 12, 34 suggests that SPC25 expression is closely related to poor prognosis in patients with HCC, non-small cell lung adenocarcinoma cells, and prostate cancer. Several studies on *in vitro* and *in vivo* functional assays of tumor cells preliminarily verified that the high SPC25 expression can promote cell proliferation in HCC and enhanced CSC properties; however, the regulatory mechanisms underlying this process remain unclear. Specific clinical sample analysis and mechanical exploration related to HCC, which is the focus of this study, are lacking.

In our study, we showed that high SPC25 expression was associated with poor prognosis in HCC and was independent of other clinical features. The expression tendency found in the clinical samples from HCC patients was consistent with earlier studies 10, 12. Moreover, high SPC25 expression was correlated with shorter OS and PFS among 142 HCC patients from our center, which increased the sample size, relative to that reported by Baozhu Zhang et al. 10. The correlation analysis also suggested that high SPC25 expression was correlated with unfavorable clinical parameters, including poor tumor differentiation, advanced TNM stage, and HBV infection. Our *in vitro* and *in vivo* studies also demonstrated that cancer cells with SPC25 overexpression were more proliferative and prone to metastasis, further confirming that SPC25 facilitated the progression of HCC.

The limited data presented in the literature showed that SPC25 upregulation in lung cancer and prostate cancer promotes the stemness of cancer cells 7, 8, suggesting a similar mechanism that allows SPC25 to facilitate tumorigenesis in HCC. Therefore, we performed the spheroid formation experiment *in vitro* to evaluate the stemness of HCC cells with SPC25 overexpression and knockdown. The results demonstrated that the HCC cells with SPC25 overexpression tended to form more and larger spheroids, whereas the cells with SPC25 knockdown showed the opposite tendency. Other biological function experiments indicated that HCC cancer cells with SPC25 overexpression exerted accelerated proliferation, improved migration and invasion, and promoted resistance to chemotherapy, thus reflecting an enhanced stemness phenotype 35. Given the challenges of stemness and invasion/metastasis experiments *in vivo,* we were not capable to provide these part of verification results, which was one of the limits of our study. Interestingly, the overexpression of SPC25 in normal hepatic cell lines could also promote the proliferation, migration and invasion but failed to facilitated spheroids formation, which suggested that the effect of SPC25 overexpression to promote cell stemness required the specific context of established HCC. More intensive explorations to explain these phenomena are needed in the future. What's more, further investigation showed that SPC25 upregulation could promote the expression of several typical stemness and EMT markers. However, there are some limitations in our study as we did not perform the corresponding *in vivo* experiments to verify its effect on stemness and migration.

Subsequently, we explored the molecular mechanism by which SPC25 promotes the proliferation and stemness of HCC. As we mentioned before, the dysregulation of SPC25 could result in abnormal cell division and cause chromosome segregation errors 13. Previous researchers has reported that chromosome segregation errors was an important cause of DNA damage. Correspondingly, we detected the level of DNA damage by the immunochemistry staining on γ-H2AX in HCC cell lines with different expression level of SPC25. Notably, the positive cell rates and staining intensity of γ-H2AX were increased in cells with SPC25 overexpression while they were decreased in cell with SPC25 silence. These results verified the increased incidence of DNA damage induced by SPC25 overexpression. Having found the phenomenon about induing DNA damage by SPC25, we next tried to explore the in-depth mechanism that regulated the biological function in HCC cells. Based on the increased DNA damage, we next detected the activation of DNA-PK, which was classically considered a component of damage response and involved in tumorigenesis 24. In addition, DNA-PK is correlated with poor prognosis independent of damage induction in numerous tumor types 36-38. We detected the phosphorylation of DNA-PK at Thr2609, and found that it was increased in HCC cells with SPC25 overexpression and decreased in HCC cells with SPC25 expression silence as we expected.

On the other hand, previous researches has revealed that both the AKT and Notch1 pathways were canonical pathways involved in tumorigenesis. Their interaction, particularly in stemness transformation, has been explained in different studies 25, 29. Accordingly, we found that both AKT and Notch1 signaling pathway were activated in HCC cells with SPC25 overexpression in our study. It was well-known that AKT signaling converts extracellular stimuli (such as receptor/ligand interaction or ligand-imposed receptor dimerization at the plasma membrane) via a phosphorylation cascade into molecular downstream responses 39. Considerable experimental evidence has clarified the interdependence between AKT signaling and Sox2-imposed CSC characteristics cooperating with Oct4, Klf4, and c-Myc in embryonic stem cells 40, 41. Combined with the results showing that the DNA-PK was activated in the same time, we speculated that there may be interaction between AKT and DNA-PK. As it was explained in many studies 42, 43, AKT forms a complex with DNA-PK by the nonhomologous end joining (NHEJ) within DNA-PK and together regulated tumor-associated processes. To further verify their specific roles in the interaction, the inhibitors of DNA-PK and AKT, AZD-7648 and MK-2206 was used in Hep3B-SPC25 and Huh7-SPC25 cells and we found that the phosphorylation of DNA-PK could not be affected by the inhibition of AKT but the phosphorylation of AKT at Ser473 was significantly suppressed by the inhibition of DNA-PK. In addition, both of the inhibitors decreased the expression of stemness markers.

With regard to the mechanism for the function of the Notch1 pathway in reprogramming stem cells has been established in numerous studies 44, 45. Notch1 signaling involves various cellular processes, such as cell proliferation and differentiation 46. Once its receptor is triggered by a ligand, Delta-Serrate-Lag2 family members are expressed on neighboring cells, followed by sequential proteolytic cleavage events in the transmembrane domain of the receptor. Consequently, the Notch intracellular domain (NICD) is released to the cytoplasm. NICD is then translocated into the nucleus, where it forms a complex with the members of the CSL transcription factor family. This complex mediates the transcription of target genes, such as Hey-2, myc, and p21 47. In their research on head and neck squamous cell carcinoma (HNSCC), Sang H Lee et al. 44 demonstrated that the constitutive activation of NICD promoted CSC traits in differentiated HNSCC cells; moreover, the downregulation of Notch1 signaling attenuated the CSC traits and enhanced the resistance of HNSCC cells to cisplatin. In our study, the expression detection of NICD and several other downstream markers revealed that the Notch1 signaling pathway was upregulated in cells with SPC25 overexpression. Currently, studies have accumulated, revealing the intertwined relationship between Notch1 and the AKT axis, particularly in cancer 25, 48. However, in these studies, Notch1 can be the upstream or downstream coordinator of the AKT pathway, rendering their mutual regulatory role unclear 29, 49, 50. In our study, the activation of Notch1 signaling was significantly inhibited by both AZD-7648 and MK-2206, but the activation of DNA-PK and AKT was not affected by the silence of Notch1in cells with SPC25. These phenomena reminded us that Notch1 was an important mediator downstream of SPC25, but the specific relationship with DNA-PK and AKT needed more verification experiments to explain, such as rescue experiments. Based on our present data, it was more reasonable to summarized that Akt signaling and Notch signaling are working in parallel. Due to the complexity of these interactions, we would perform rescue and other in-depth experiments to explain the concrete regulation interaction among DNA-PK, AKT and Notch1 in our future researches.

Subsequently, we aimed to explore the biological effect in HCC cells after the blockade of DNA-PK/AKT/Notch1. From results in Fig. 6, we could summarize that the abilities of stemness, proliferation, migration and invasion in HCC cells with SPC25 overexpression were significantly suppressed by AZD-7648, MK-2202 and the silence of Notch1, which respectively inhibited the activation of DNA-PK, AKT and Notch. Taken together, we could draw a deduction that DNA-PK, AKT, and Notch1 signaling pathways might be the critical mechanism that promoted the tumor growth and progression in HCC cells with SPC25 upregulation.

In summary, we elucidated that increased SPC25 expression in HCC was closely correlated with poor prognosis. SPC25 functions as an oncogene related to the regulation of tumor cell stemness to promote HCC progression via the DNA-PK/AKT/Notch1 pathways whose detailed relationships remain to be determined and blockade of these pathways suppressed the tumorigenic effect induced by SPC25 upregulation. Therefore, our study provided a potential therapeutic target for HCC intervention. Future studies to determine the in-depth mechanisms underlying SPC25 upregulation may present new insights into HCC treatment.

## Supplementary Material

Supplementary figures.

## Figures and Tables

**Table 1:** Association of SPC25 expression with clinicopathological features in patients with HCC

Characteristic	Total	SPC25 expression (n=142)		*χ^2^*	*P*-value
		Negative/low n=71 (50.0%)	High n=71 (50.0%)		
**Age, years**					
≥50	66	34	32	0.11	0.736
<50	76	37	39		
**Gender**					
Male	126	60	66	2.54	0.111
Female	16	11	5		
HBsAg					
Positive	126	59	67	4.508	**0.034**
Negative	16	12	4		
**Liver cirrhosis**					
Yes	68	30	38	1.805	0.179
No	74	41	33		
**Tumor size**					
≥5cm	88	43	45	0.120	0.730
<5cm	54	28	26		
**Tumor number**					
Multiple	31	11	20	3.343	0.068
Single	111	60	111		
**AFP**					
≥400ng/ml	53	21	32	3.643	0.056
<400ng/ml	89	50	39		
**Histological differentiation**					
Well	30	22	8	8.283	**0.004**
Poor	112	49	63		
**Tumor encapsulation**					
Yes	59	32	27	0.725	0.395
No	83	39	44		
**TNM stage**					
I	90	51	39	4.369	**0.037**
II or III	52	20	32		
**Vascular invasion**					
Yes	20	6	14	3.733	0.053
No	120	64	56		

**Table 2:** Univariate and multivariate analyses of factors associated with overall survival and progression free survival

Variable	Overall survival				Progression free survival			
	Univariate cox		Multivariate cox		Univariate cox		Multivariate cox	
	*P*-value	HR (95%CI)	*P*-value	HR (95%CI)	*P*-value	HR (95%CI)	*P*-value	HR (95%CI)
**Age**								
≥50 or <50	0.3267	0.78 (0.47-1.28)			0.1016	0.70 (0.45-1.07)		
**Gender**								
Male or female	0.2619	1.69 (0.68-4.2)			0.8706	0.95 (0.50-1.79)		
**HBsAg**								
Positive or negative	0.5504	1.29 (0.56-3.00)			0.7793	0.91 (0.47-1.76)		
**Liver cirrhosis**								
Yes or no	0.0429	1.67 (1.02-2.74)			0.3898	1.20 (0.79-1.84)		
**Tumor size**								
≥5 cm or <5 cm	**0.0022**	2.43 (1.38-4.29)	**0.0032**	2.39 (1.34-4.25)	**0.0010**	2.23 (1.38-3.61)	**0.0013**	2.23 (1.37-3.63)
**TNM stage**								
I or II/III	**<0.0001**	3.39 (2.06-5.58)	**<0.0001**	3.18 (1.89-5.33)	**<0.0001**	3.13 (2.03-4.80)	**<0.0001**	3.53 (2.25-5.41)
**Tumor encapsulation**								
Yes or no	0.3270	1.28 (0.78-2.09)	**0.0173**	1.85 (1.11-3.07)	0.3008	1.25 (0.82-1.91)	**0.0200**	1.70 (1.09-2.64)
**Tumor number**								
Single or multiple	**<0.0001**	3.11 (1.84-5.23)			**<0.0001**	2.75 (1.74-4.37)		
**AFP**								
≥400 ng/ml or <400 ng/ml	0.9978	1.00 (0.60-1.68)			0.1977	1.33 (0.86-2.05)		
**Histological differentiation**								
Well or poor	0.3490	0.74 (0.40-1.39)			0.0631	1.72 (0.97-3.05)		
**Vascular invasion**								
Yes or no	**0.0317**	1.99 (1.06-3.74)			**0.02780**	1.87 (1.07-3.27)		
**SPC25**								
High or low	**0.0076**	1.99 (1.20-3.31)	**0.0180**	1.87 (1.11-3.15)	0.1419	1.40 (0.89-2.20)	**0.0451**	1.60 (1.01-2.55)
