# Supplementary material for: SPC25 promotes proliferation and stemness of hepatocellular carcinoma cells via the DNA-PK/AKT/Notch1 signaling pathway
Source: Int J Biol Sci. 2022 Aug 15;18(14):5241–59. doi: 10.7150/ijbs.71694 (PMC9461674; doi:10.7150/ijbs.71694)
Supplement: Supplementary file 1 — Supplementary figures. [file ijbsv18p5241s1.pdf]

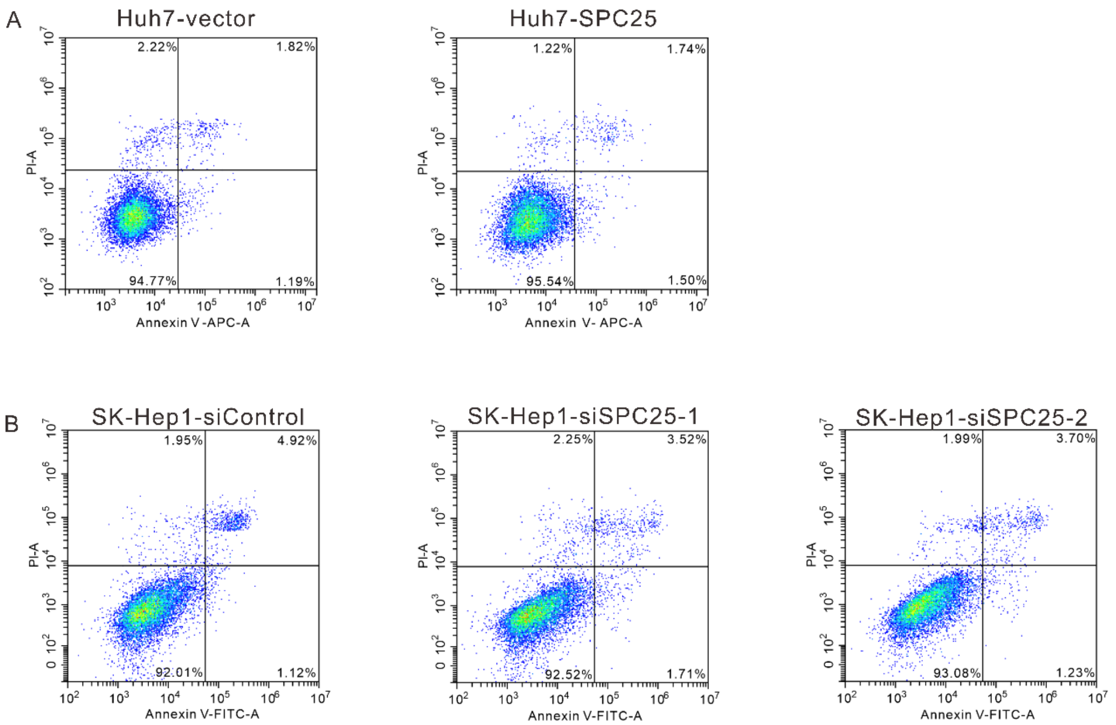

Supplementary Figure 1. The apoptosis assay performed in Huh7-vector and Huh7-SPC25 cells (A) and SK-Hep1-siControl, SK-Hep1-siSPC25-1, and SK-Hep1-siSPC25-2 cells (B). The Annexin V-APC positive cells represented the sum of apoptotic cells.

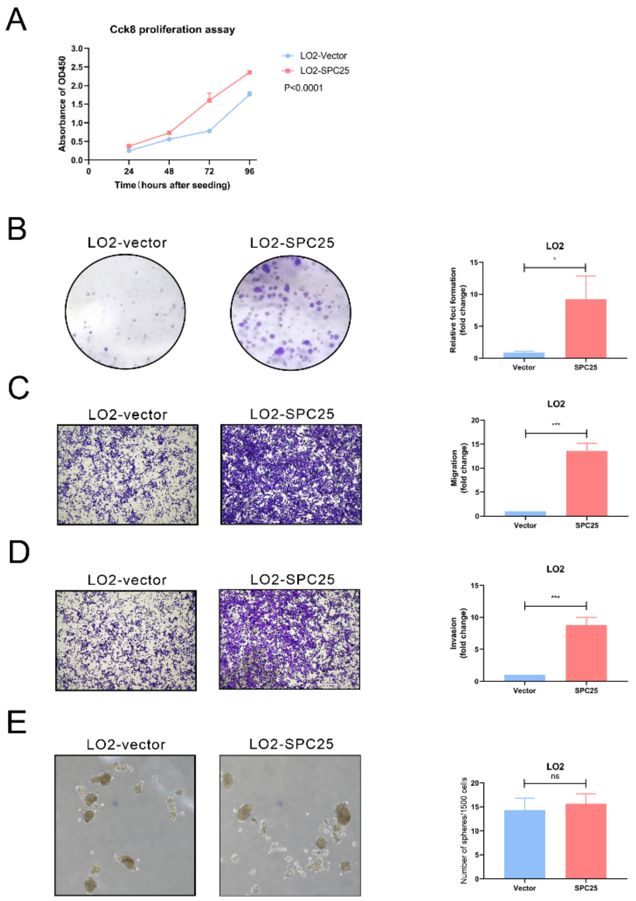

Supplementary Figure 2. A serial of biological function assays performed in LO2-vector and LO2-SPC25 cells. (A) Cck8 proliferation assay performed 72 hours after the transfection with different plasmids. (B) Colony formation assay demonstrated the cell colonies incubated for 14 days. (C) Transwell migration assay showed the number of migrated cells from the upper to the bottom chamber 24 hours after the seeding. (C) Invasion assay showed the number of migrated cells from the upper chamber coated with Matrigel to the bottom chamber 24 hours after the seeding. (E) Spheroid formation assay demonstrated the spheres formed by LO2-vector cells and LO2-SPC25 cells for 7 days.

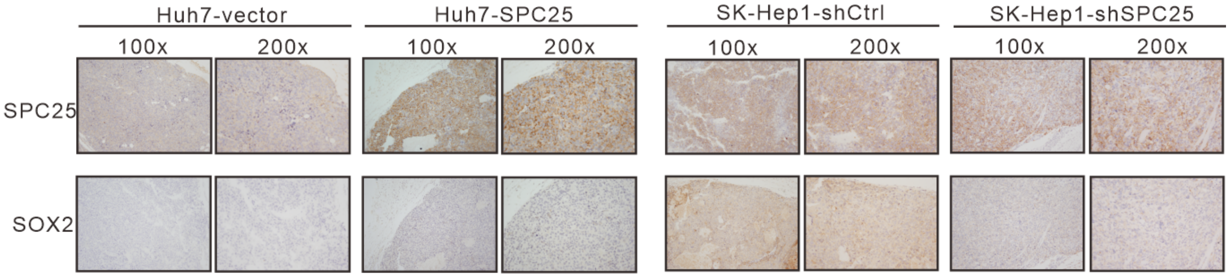

Supplementary Figure 3. Representative images of IHC staining showing the expression level of SPC25 and Sox2 in tissues slices from NCG mice transplanted with Huh7 and SK-Hep1 cells transfected with the indicated constructs.

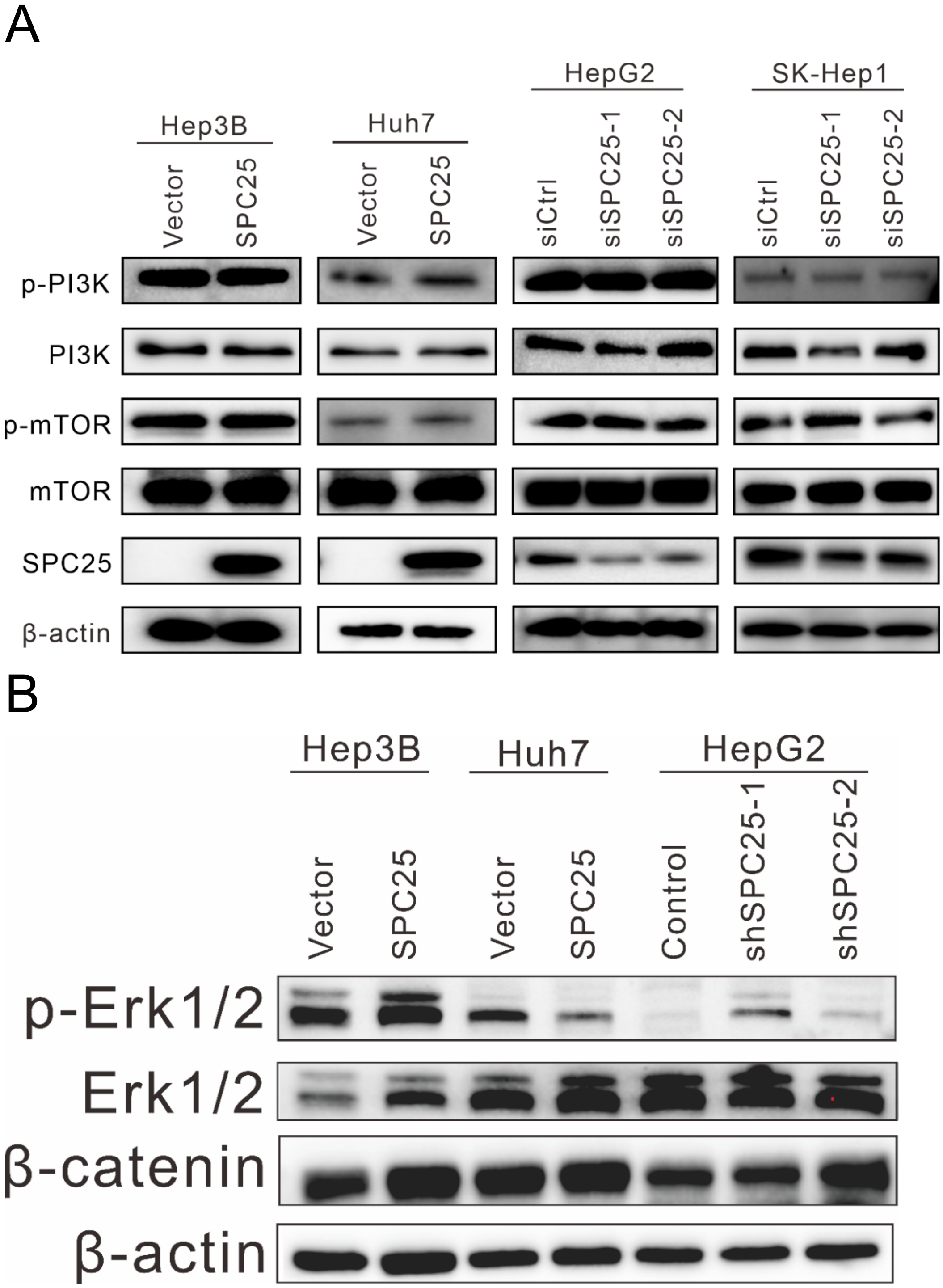

Supplementary Figure 4. (A) Western blot showed the activation status of PI3K and mTOR. (B) The status of ERK1/2 and Wnt/β-catenin pathway in Huh7, Hep3B and HepG2 cells transfected with the indicated constructs.

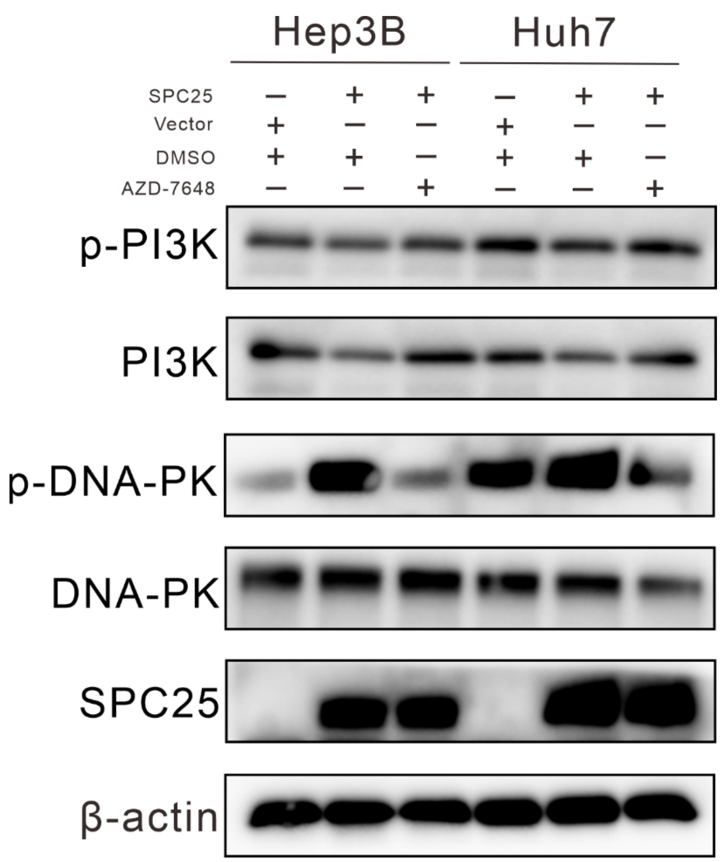

Supplementary Figure 5. Western blot showed the activation status of PI3K and DNA-PK in indicated Hep3B and Huh7 cells after the treatment of 5μM AZD-7648.
